# Growth Associated Protein 43 (GAP-43) as a Novel Target for the Diagnosis, Treatment and Prevention of Epileptogenesis

**DOI:** 10.1038/s41598-017-17377-z

**Published:** 2017-12-18

**Authors:** Ashley D. Nemes, Katayoun Ayasoufi, Zhong Ying, Qi-Gang Zhou, Hoonkyo Suh, Imad M. Najm

**Affiliations:** 1 Epilepsy Center, Neurological Institute, Cleveland Clinic, 44195 Cleveland, OH USA; 2 Department of Immunology, Lerner Research Institute, Cleveland Clinic, Cleveland, OH USA; 3 Department of Stem Cell Biology and Regenerative Medicine, Lerner Research Institute, Cleveland Clinic, Cleveland, OH USA

## Abstract

We previously showed increased growth associated protein 43 (GAP-43) expression in brain samples resected from patients with cortical dysplasia (CD), which was correlated with duration of epilepsy. Here, we used a rat model of CD to examine the regulation of GAP-43 in the brain and serum over the course of epileptogenesis. Baseline GAP-43 expression was higher in CD animals compared to control non-CD rats. An acute seizure increased GAP-43 expression in both CD and control rats. However, GAP-43 expression decreased by day 15 post-seizure in control rats, which did not develop spontaneous seizures. In contrast, GAP-43 remained up-regulated in CD rats, and over 50% developed chronic epilepsy with increased GAP-43 levels in their serum. GAP-43 protein was primarily located in excitatory neurons, suggesting its functional significance in epileptogenesis. Inhibition of GAP-43 expression by shRNA significantly reduced seizure duration and severity in CD rats after acute seizures with subsequent reduction in interictal spiking. Serum GAP-43 levels were significantly higher in CD rats that developed spontaneous seizures. Together, these results suggest GAP-43 as a key factor promoting epileptogenesis, a possible therapeutic target for treatment of progressive epilepsy and a potential biomarker for epilepsy progression in CD.

## Introduction

Epilepsy is the fourth most common neurological disorder in the United States^1,2^. About 30–40% of patients are not responsive to pharmacological treatment and only ~47% of patients will remain seizure-free 10 years after surgical resection^3,4^. Seizure freedom after surgery is negatively correlated with duration of epilepsy, such that the longer the disease duration, the worse the outcome is after surgery^5^. In order to develop new treatments for the prevention and cure of epilepsy, it is important to understand mechanisms of disease development and progression.

Congenital focal brain malformations known as cortical dysplasia (CD) are common pathological substrates of medically intractable epilepsies. Patients with CD may not develop epilepsy until the occurrence of the first seizure in the setting of a “trigger”; a phenomenon we refer to as a “second hit” (e.g. sleep deprivation, minor head trauma, brain infection, cerebral ischemia…)^6–11^. The first seizure is typically followed by a latent period, leading to seizure recurrence and the development of drug resistance in some patients. These observations suggest that epilepsy is a non-static disease, and the epileptic brain region may continue to undergo progressive cellular and molecular changes over time.

There is increasing evidence that cellular and neural networks at the local epileptic area extend beyond the lesion, which may explain why some surgical resections classically fail^12,13^. We have recently shown that the duration of epilepsy in patients with CD is correlated with increased expression of growth-associated protein 43 (GAP-43), a marker of axonal and synaptic growth^14^. GAP-43 is upregulated following stroke and traumatic brain injury^15–19^. Our results suggest the interesting possibility that GAP-43 is upregulated after a second hit (e.g. seizure), leading to the formation of highly interconnected, synchronized epileptic neural networks. However, studying surgically resected human brain specimen has a significant limitation for the investigation of the dynamic changes of GAP-43 during epileptogenesis. Therefore, we investigated the role of GAP-43 expression in epileptogenesis using a well-characterized animal model of CD.

Our study shows that GAP-43 expression is located predominantly within excitatory neurons, and its expression is increased in brains after an acute seizure. GAP-43 in serum is also increased in CD rats that developed spontaneous epilepsy. Knockdown of GAP-43 significantly reduced epileptogenesis and epileptogenicity in CD rats. The findings presented in this study provide valuable insight into some possible molecular mechanisms of epileptogenesis and identify GAP-43 as a potential new target protein for the treatment and prevention of epilepsy in cortical dysplasia.

## Results

### CD animals exhibited more severe seizures after PTZ treatment

**Figure 1:**
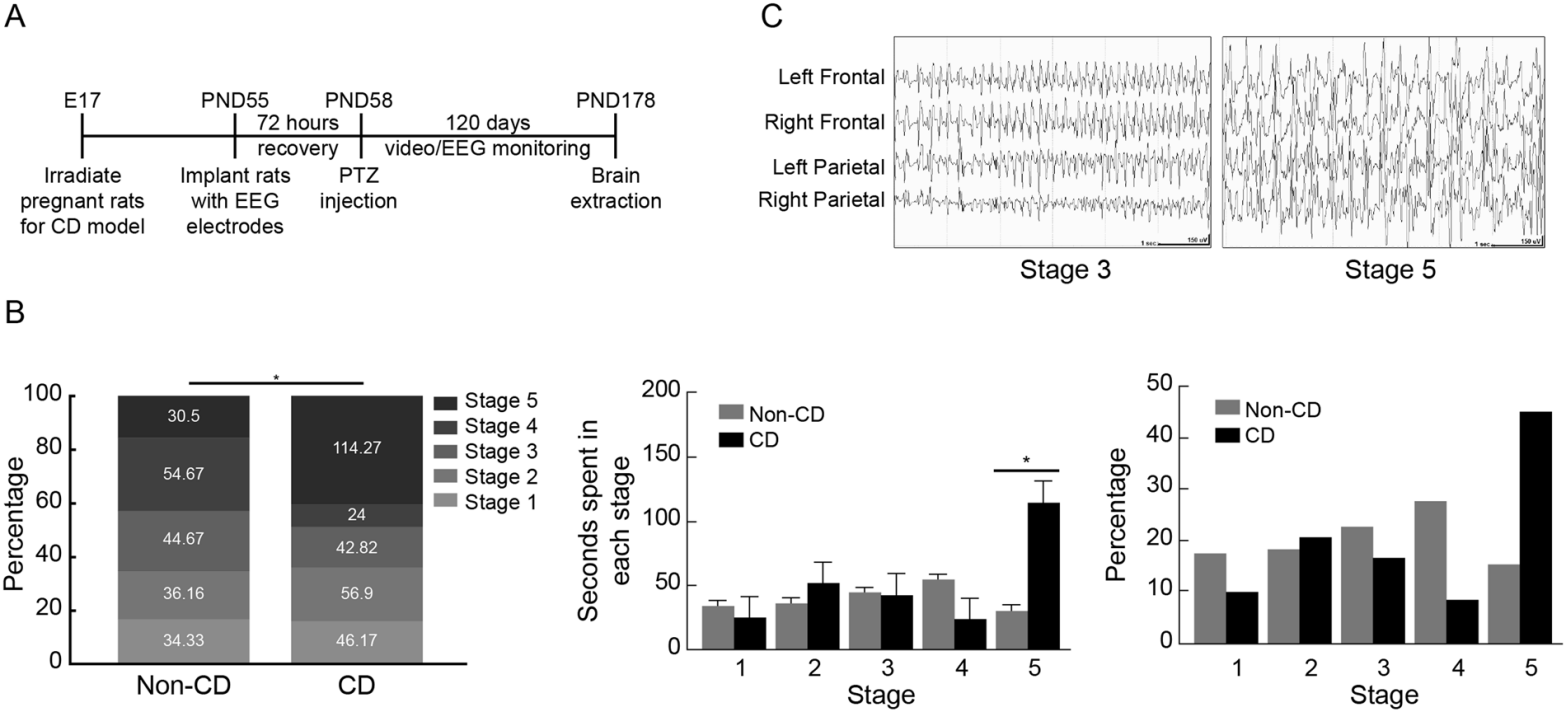
Chronic epilepsy in an experimental rat model of Cortical Dysplasia. (**A**) Timeline of experimental procedures to study epileptogenesis in cortical dysplasia (CD) and non-CD control rats after Pentylenetetrazole (PTZ) induced acute seizures. (**B**) (Left) Percentage of total seizure duration spent in each stage of seizure severity based on Racine’s scale (Racine, 1972). Duration is expressed in seconds (numbers inside of bars, and separately in middle graph) and percent of total seizure duration (also shown in right graph separately). (**C**) Examples of EEG observed in stage 3 (left) and stage 5 (right) of seizure severity after PTZ injection. Error bars show standard error of the mean. Sample sizes: Non-CD + Saline n = 6, Non-CD + PTZ n = 8, CD + Saline n = 7, CD + PTZ n = 8.

Consistent with previous studies, CD brains were smaller, with a thinner cortex, hippocampi had heterotopic bursts in CA1 and CA2 areas, enlarged ventricles, and disorganized cortical architecture similar to human focal cortical dysplasia (FCD) type I^20–24^. Only implanted rats (non-CD + Saline n = 6; non-CD + PTZ n = 8; CD + Saline n = 7; CD + PTZ n = 8) were used for EEG analyses (Fig. 1A). All CD rats developed severe acute seizures (100%) following the systemic administration of a subconvulsive dose of PTZ (40 mg/kg), while non-CD control rats displayed mild (53.33%) or no seizures using the same PTZ dose. Of the animals that had seizures, CD rats had a mean ictal duration of 136.9 sec, and non-dysplastic rats had a mean ictal duration of 71.29 sec (n.s., p = 0.289). Each seizure was graded using Racine’s Scale^25^ and the duration spent in each grade was analyzed across groups with a mixed models regression analysis and Tukey-Kramer adjustments. CD rats spent more time in the most severe stage (stage 5), displaying generalized tonic-clonic behaviors, while seizures in control rats were less severe, and equally distributed across all stages of behavioral severity (Fig. 1B and C; p = 0.025). Overall, with the same injected dose of PTZ, CD rats exhibited longer, more severe seizures than non-CD animals.

**Figure 2:**
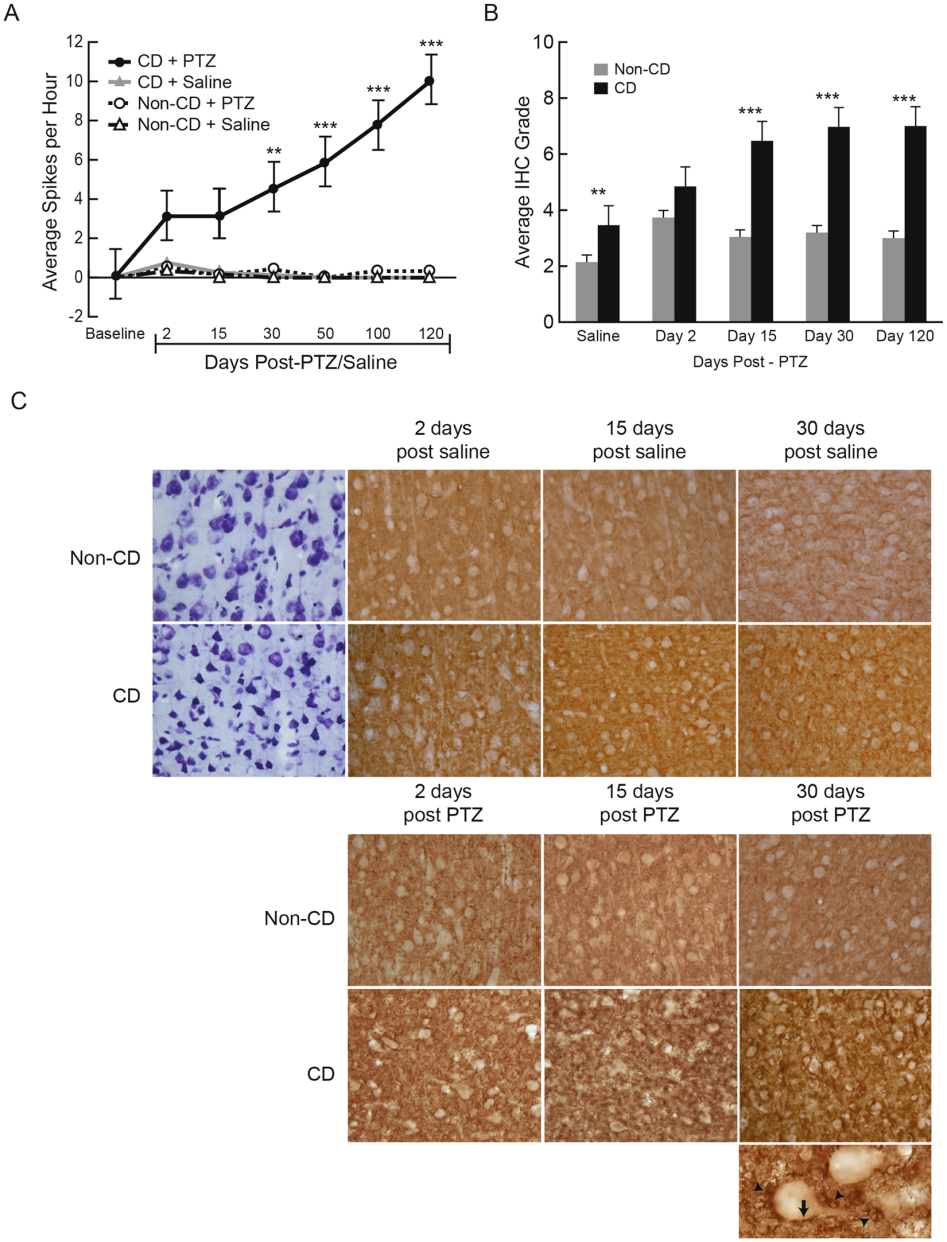
GAP-43 protein expression over the development of epilepsy. (**A**) Interictal spike frequency over 120 days after PTZ induced acute seizure, or saline injection. Sample sizes for EEG: Non-CD + Saline n =   6, Non-CD + PTZ n = 8; CD + Saline n = 7; CD + PTZ n = 8. (**B**) Qualitatively graded immunohistochemistry (IHC) shows expression of GAP-43 staining in the dysplastic cortex and non- CD cortex over time after an acute seizure. Error bars show standard error of the mean. (**C**) Representative images of GAP-43 IHC at different time points. In PTZ injected groups, CD rats show progressively increased GAP-43 immunostaining over time. The GAP-43 immunostaining patterns are expressed as rim structures surrounding the cell body (arrows) and tubular patterns (arrowheads) illustrated in the bottom image at 63x magnification. Sample sizes for IHC: Non-CD + Saline n = 11, + PTZ Day 2 n = 11, + PTZ Day 15 n = 7, + PTZ Day 30 n = 9, + PTZ Day 120 n = 5. CD + Saline n = 8, + PTZ Day 2 n = 8, + PTZ Day 15 n = 8, + PTZ Day 30 n = 7, + PTZ Day 120 n = 5.

All CD + PTZ rats exhibited increasing interictal spiking after the initial acute seizure induced with PTZ (Fig. 2A). After 120 days, three out of five (60%) CD + PTZ rats developed spontaneous seizures (SS). None of the non-CD rats (saline or PTZ-treated) developed spontaneous epileptic activity. CD rats with SS had the highest frequency of interictal spikes over 120 days when compared to all other groups (p = 0.047). Consistent with our previous findings using this model, saline-treated animals did not have seizures, nor exhibited any abnormal behavior or epileptic (ictal or interictal) EEG patterns^10,21–23^.

### GAP-43 expression is higher in CD brains and is up-regulated after an acute seizure

There was no significant effect from surgical implantation of EEG electrodes on GAP-43 expression, therefore rats were pooled for GAP-43 analyses (n.s. p = 0.47; Histology: n = 20 non-CD, n = 18 CD; Molecular biology: n = 28 non-CD, n = 27 CD). Sections stained with immunohistochemistry (IHC) for GAP-43 protein were randomized and qualitatively graded as previously described^14^. Bilateral somatosensory cortex images (A/P = −2.0 ± 0.2 mm) were taken and analyzed (two coronal slices, for a total of 4 images per rat). Each field of view contained 53.5 cells (SEM = 4.17). There was no difference in the number of cells imaged in CD and non-CD brains regardless of the architectural differences. The IHC staining intensity in the cortex was given a grade 2–8 based on neuronal rim staining (cell surface) and tubular punctate expression (intracellular) by two blinded investigators (AN and ZY). As previously reported, the scores were calculated based on the sum of qualitative grading of Rim and Tubular Punctate GAP-43 expression^14^. Rim Staining Value: 4 = more than 50% of neurons in the image positive for GAP-43 stained rim, 3 = more than 10% but less than 50%, 2 = about 5–10%, 1 = less than 5% of neurons; Tubular Punctate Staining Value: 4 = intensely dark, 3 = moderately dark, 2 = some punctate staining, 1 = no staining.

A mixed models regression analysis was conducted on GAP-43 immunohistochemistry staining with Tukey-Kramer adjustments. There was no change in GAP-43 expression over time (up to 120 days following saline treatment) within CD (p = 0.381) and non-CD (p = 0.252) groups, suggesting that normal brain maturity did not affect GAP-43 expression. Therefore, data were pooled into CD and non-CD saline groups. CD rats treated with saline had significantly higher GAP-43 expression than non-CD rats with saline, showing that CD pathology alone is associated with a baseline increase in GAP-43 levels (Fig. 2B and C, p = 0.007). CD rats treated with PTZ also had significantly higher GAP-43 expression as compared to CD rats treated with saline, showing a change in protein expression in response to an acutely induced seizure (p = 0.0001). GAP-43 expression was further increased overall in CD brains after PTZ induced seizures, with the greatest differences compared to non-CD animals at day 15 (p = 0.0008), day 30 (p = 0.0009) and day 120 (p = 0.001).

**Figure 3:**
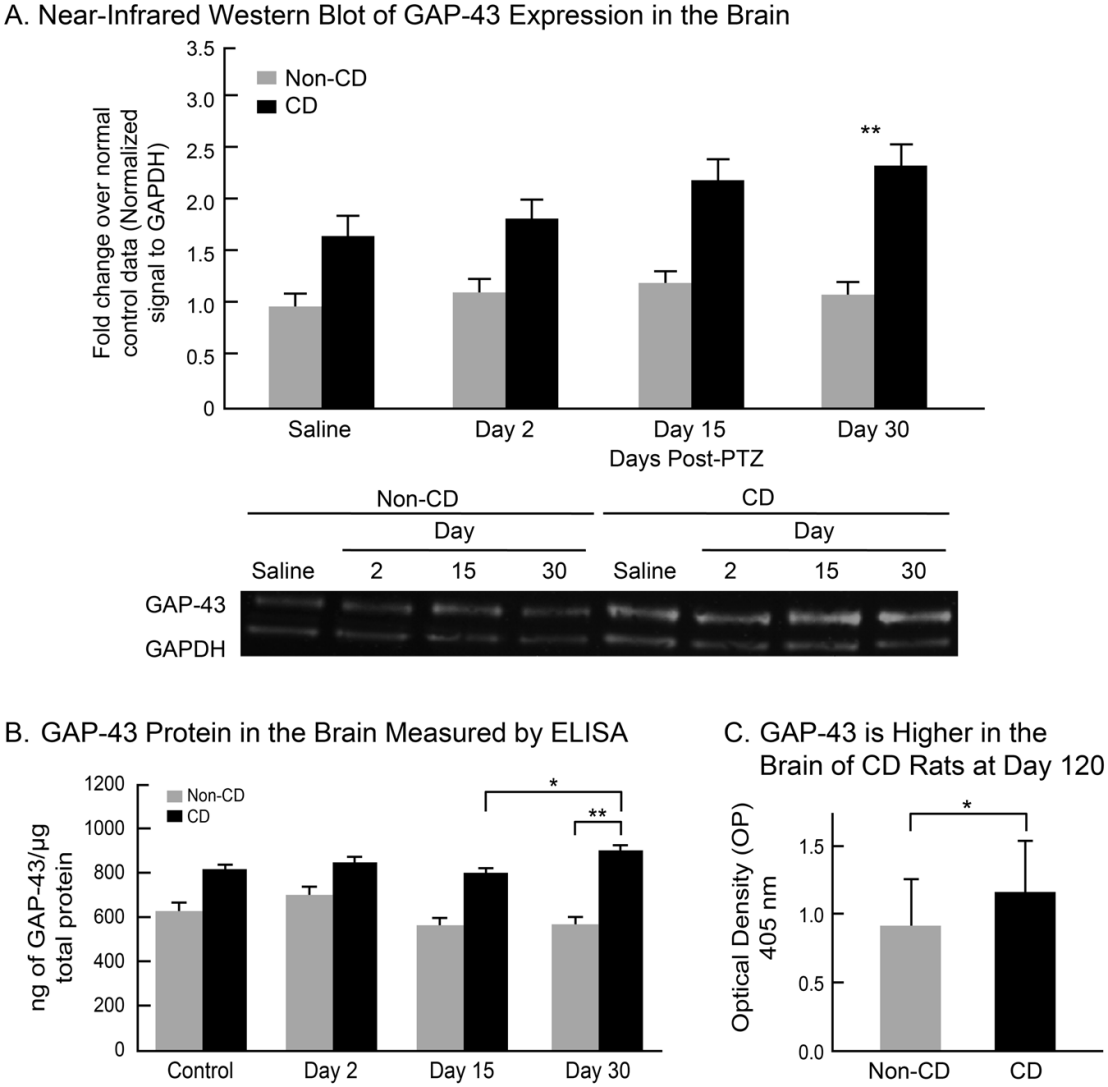
Quantified GAP-43 protein expression in the brain. (**A**) Near-infrared (NIF) western blots normalized to GAPDH show GAP-43 protein in CD and non-CD brains at each time point after PTZ induced seizures. Western blot image was cropped to remove protein ladder. (**B**) GAP-43 protein in the brain as measured by ELISA in CD rats compared to non-CD rats at each time point. (**C**) ELISA results show a higher level of GAP-43 protein in CD rats as compared to non-CD rats 120 days after PTZ induced acute seizures. Error bars represent standard error of the mean. Sample sizes: Non-CD + Saline n = 5, + PTZ Day 2 n = 4, + PTZ Day 15 n = 4, + PTZ Day 30 n = 4. CD + Saline n = 4, + PTZ Day 2 n = 4, + PTZ Day 15 n = 4, + PTZ Day 30 n = 4.

Quantified results of ELISA and NIR western blotting were analyzed with mixed models regression analyses testing measured response against group, day, and group-day interaction. Tukey-Kramer adjustments were made for all pairwise comparisons. Similar to the IHC observations, NIR western blotting results showed an increase in GAP-43 protein level at day 30 in CD rats as compared to non-CD rats (Fig. 3A, p = 0.001). Non-CD brains showed no change in GAP-43 protein levels throughout the experiment. Further supporting these findings, ELISA results showed heightened levels of GAP-43 protein in CD brains at days 15 and 30 (p = 0.019), while non-CD brains remained stable at each time point (Fig. 2B). CD rats had higher GAP-43 protein than non-CD rats at day 120 (Fig. 2C, p = 0.04). There was no difference in GAP-43 protein expression between CD rats that developed spontaneous epilepsy compared to CD rats that only exhibited interictal spikes (p = 0.45). GAP-43 expression was correlated with interictal spike frequency (R^2^ = 0.8649, p = 0.01), suggesting a possible relationship between the appearance and recurrence of spikes and GAP-43 upregulation.

**Figure 4:**
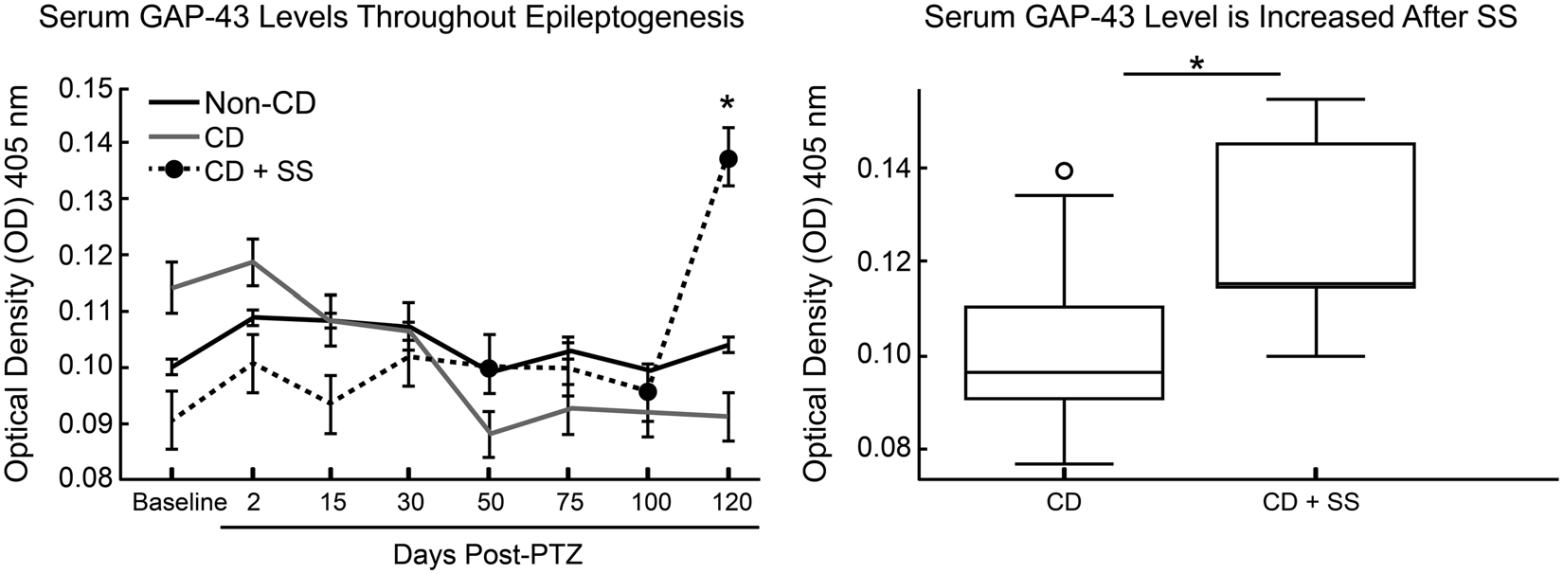
Serum GAP-43 as a biomarker of epilepsy progression. Levels of serum GAP-43 (left) over 120 days after PTZ induced acute seizures. Pooled data (right) from individual time points of rats with CD that did not have spontaneous seizures compared to the time points in which CD rats were chronically epileptic with spontaneous seizures (CD + SS). Error bars represent standard error of the mean. Sample sizes: Non-CD n = 20, CD n = 18, CD + SS n = 3.

Remarkably, serum GAP-43 levels were significantly higher in CD rats after SS (Day 120) as compared to CD rats without SS (p = 0.028), and non-CD rats (p = 0.015) (Fig. 4). A Pearson correlation coefficient was calculated to determine the relationship between serum GAP-43 and epileptic activity. Serum GAP-43 levels were not correlated with GAP-43 expression in the brain (R^2^ = 0.04, n.s. p = 0.63). Interestingly, serum GAP-43 levels were correlated with interictal spikes at days 100 and 120 (R^2^ = 0.29, p = 0.05). Although further research is necessary to determine the mechanisms of serum GAP-43 upregulation with seizures, GAP-43 protein was found localized to capillary lumen in the CD + SS rat brain, suggesting that it may be released into the blood after synapse formation. These results support the notions of a seizure-induced breakdown of the blood brain barrier^26,27^. Further research is necessary to determine the mechanisms and timing of GAP-43 increase in the serum throughout the development of SS.

### GAP-43 is mainly expressed in excitatory synapses

**Figure 5:**
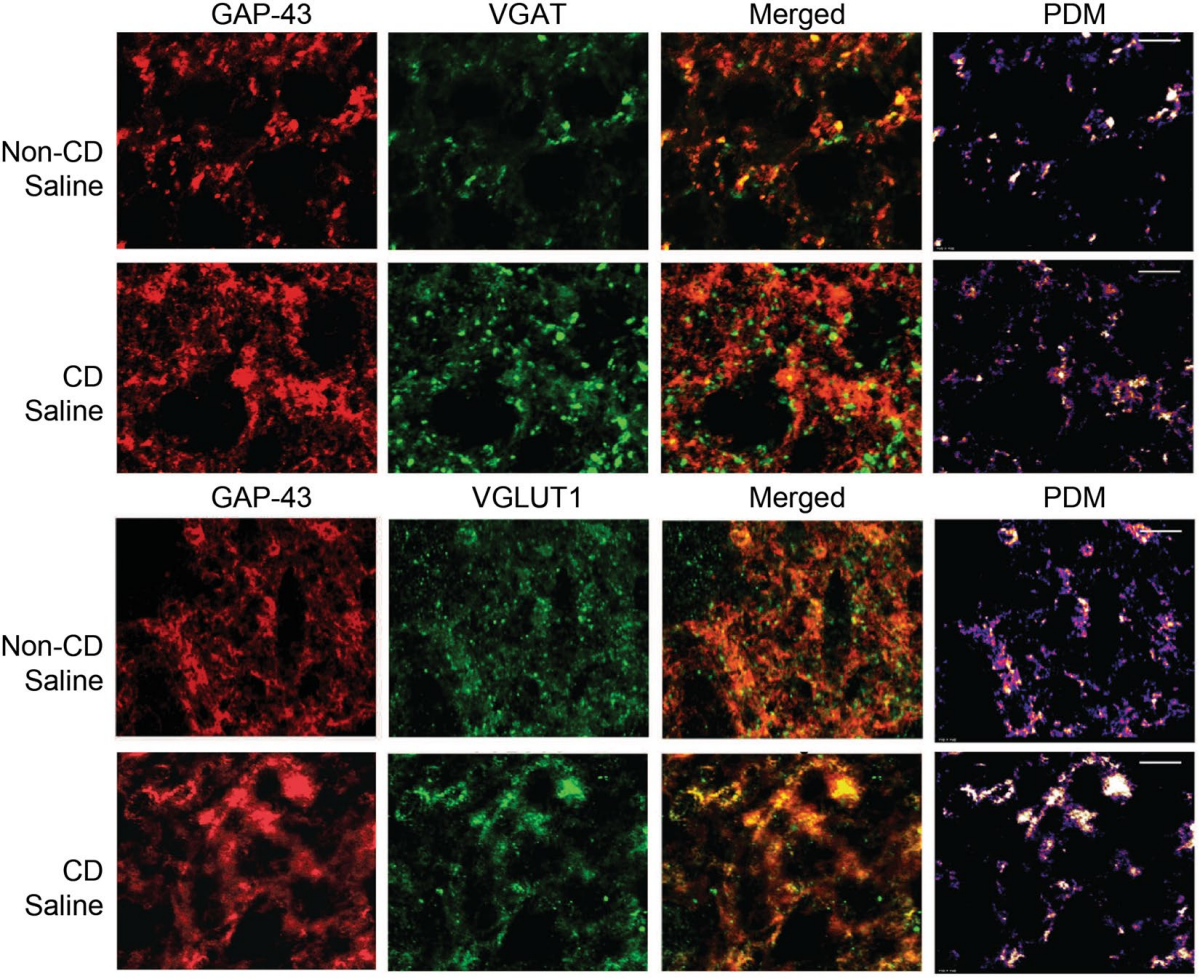
Co-localization of GAP-43 with presynaptic markers of inhibition and excitation. Confocal images of cortical tissue stained for GAP-43 (red) with VGAT (green; top) and VGLUT1 (green; bottom). Product of the Differences from the Mean (PDM) images show areas where both images express co-localization with pixels above the mean intensity. PDM = (red intensity - mean red intensity) × (green intensity - mean green intensity) as described in Li **et al**. 2004. Scale bar = 5 μm. Sample sizes: non-CD n = 4, CD n = 4.

**Figure 6:**
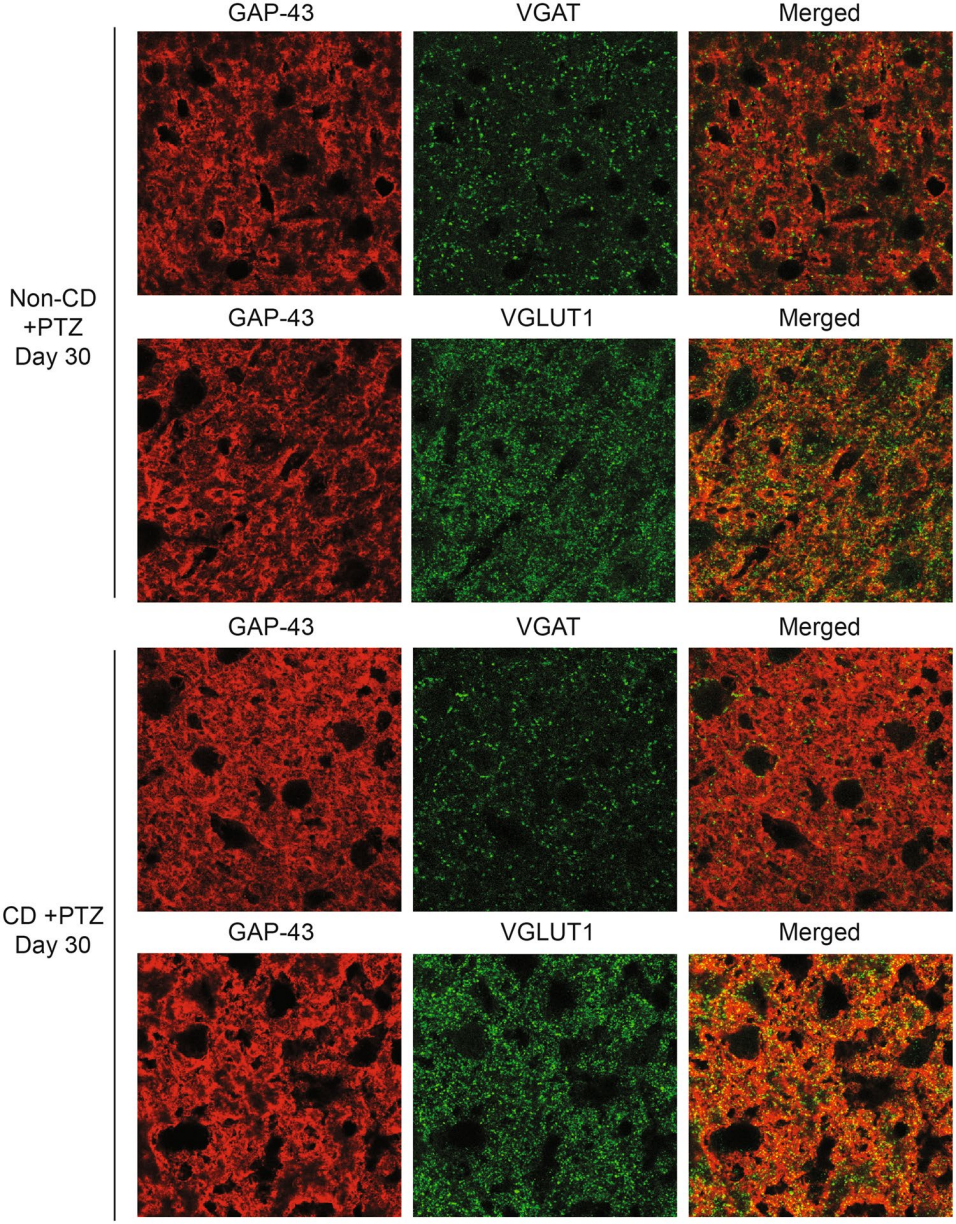
Co-localization of GAP-43 with presynaptic markers of inhibition and excitation 30 days after PTZ induced seizures. Confocal images of cortical tissue stained for GAP-43 (red) with VGAT (green; top) and VGLUT1 (green; bottom) from non-CD (n = 4) and CD (n = 4) animals 30 days after PTZ induced acute seizures. Scale bar = 50 μm.

**Figure 7:**
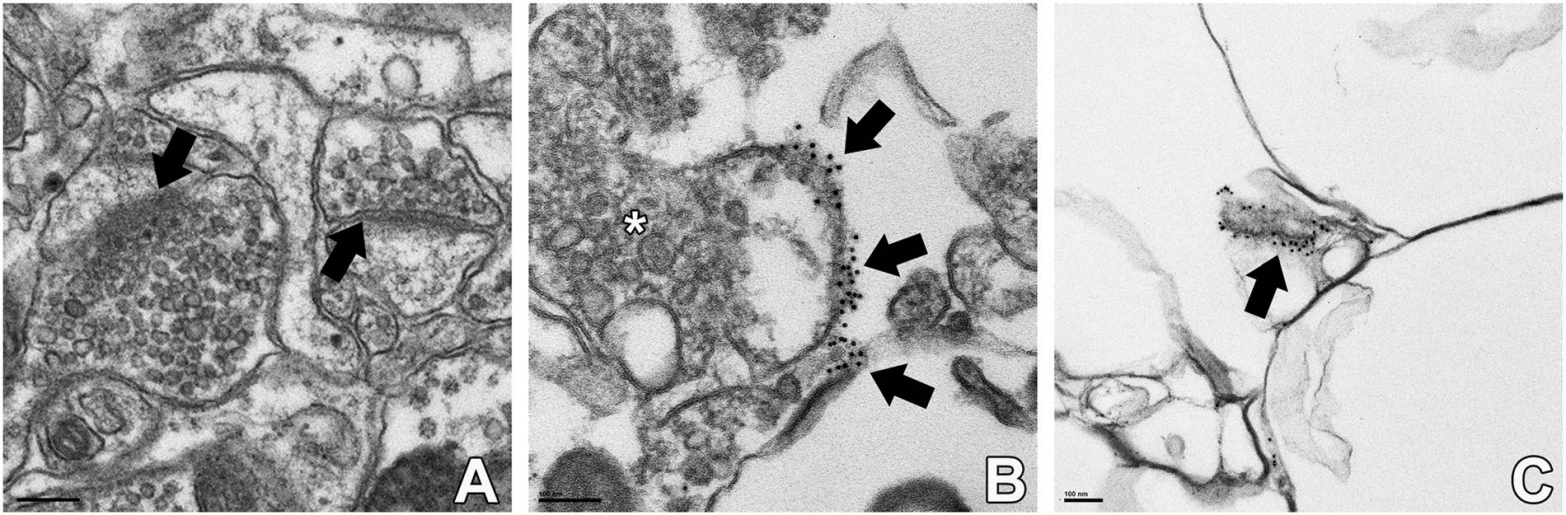
Immuno-electron microscopy. (**A**) Mature excitatory and inhibitory synapses show absence of GAP-43 signal in the cortex of a Non-CD rat 4 months after PTZ induced acute seizure. The left arrow shows an excitatory synapse with spherical vesicles and asymmetric postsynaptic density. The right arrow shoes an inhibitory synapse with flattened vesicles and symmetric postsynaptic density. (**B**) Axon terminal within the cortex of a CD rat 4 months after PTZ induced acute seizure. Arrows show GAP-43 (10 nm gold). The star (*) shows spherical vesicles within the axon suggestive of excitatory output as described in Gray, 1959. (**C**) Capillary lumen from a CD animal with spontaneous seizures shows GAP-43 positive staining. Scale bars = 100 nm. Sample sizes: CD + PTZ n = 2, Non-CD + PTZ n = 2.

In order to determine if GAP-43 positive synapses are primarily excitatory or inhibitory, we performed co-localization analyses with immunofluorescent staining of brain sections from non-CD and CD animals treated with saline or PTZ, 30 days post-treatment (Figs 5 and 6). The data were analyzed for pixel intensity using a two-way ANOVA and Tukey’s post-hoc test. Co-localization was analyzed using ImageJ to calculate Pearson’s correlation coefficient as described^28^. With saline, CD brains had a significantly lower pixel intensity in VGAT stained sections (p = 0.04), and a significantly higher pixel intensity in GAP-43 stained sections (p = 0.01) than non-CD brains. There was no significant difference in VGLUT1 pixel intensity (n.s. p = 0.07). Double-labeled images showed a stronger co-localization between GAP-43 and VGLUT1 in both CD and non-CD brains (p = 0.005). The subcellular localization of GAP-43 within axons containing spherical vesicles was confirmed by immuno-EM staining (Fig. 7A and B). These results further suggest a role of GAP-43 in the formation of excitatory synapses^29^.

### Knockdown of GAP-43 Reduces Epileptogenicity

**Figure 8:**
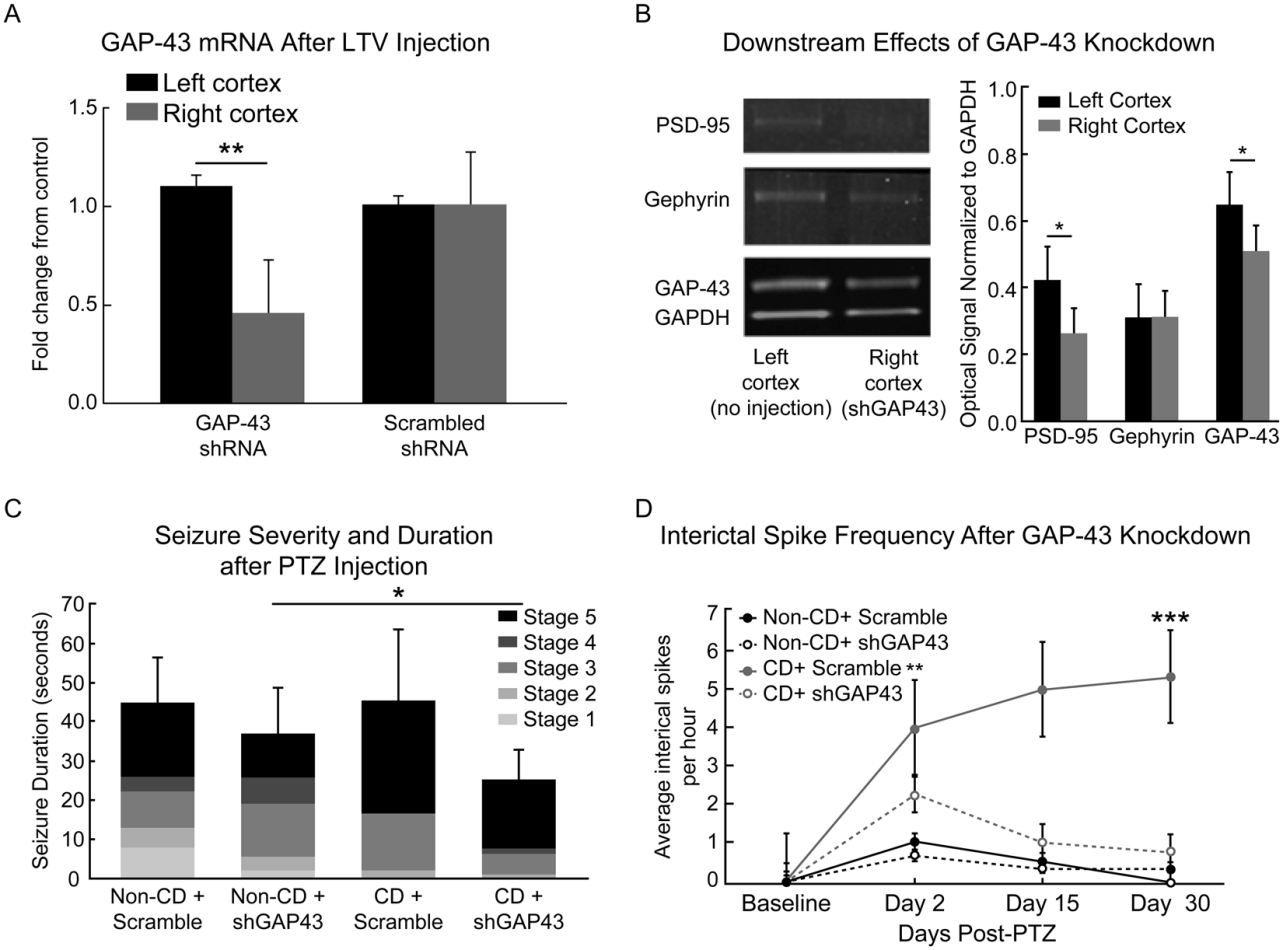
The effects of GAP-43 knockdown on epileptogenesis. (**A**) RNA analysis of GAP-43 RNA in the right and left cortices. The lentiviral vector (LTV) containing shRNA against GAP-43 (shGAP43), or a scrambled non-target control sequence (Scramble), was injected into the right cortex (N = 4). (**B**) NIR western blots from left and right cortices with shGAP43 LTV injection into the right cortex (N = 4). Western blot images were cropped to show the protein changes in one representative animal. Images shown were collected from the same gel at different molecular weights (see Supplementary Figure). (**C**) Seizure severity graded with Racine’s scale after PTZ induced seizures in LTV rats with shGAP43 or Scramble control sequence (Racine, 1972). (**D**) Interictal spike frequency before and after PTZ induced acute seizure in CD and non-CD rats with LTV injections of either shGAP43 or Scramble. Error bars show standard error of the mean. Sample sizes for Seizure Duration and Spike Frequency: Non-CD + Scramble n = 3, Non-CD + shGAP43 n = 4, CD + Scramble n = 3, CD + shGAP43 n = 4.

Mixed models regression analysis testing with Tukey-Kramer adjustments were used to determine the changes after lentiviral (LTV) knockdown. Animals which received a LTV vector containing shRNA against GAP-43 (shGAP43) injected into the right somatosensory cortex, showed ~50% reduction in local GAP-43 RNA (p = 0.016, Fig. 8A). GAP-43 protein expression was reduced in the right hemisphere of knockdown brains (p = 0.04; Fig. 8B). PSD-95 protein was also reduced in the right cortex of knockdown brains (p = 0.02). Treatment with a scrambled control sequence (Scramble) did not change GAP-43 RNA or protein expression. LTV knockdown of GAP-43 did not affect PTZ-seizure thresholds (mean thresholds: non-CD + shGAP43 = 56.25 mg/kg; non-CD + scramble = 63.33 mg/kg; CD + shGAP43 = 47.5 mg/kg; CD + scramble = 51.67 mg/kg; n.s. p = 0.48). More CD + shGAP43 rats reached a peak stage 5 in seizure severity than non-CD rats with shGAP-43 (p = 0.04; Fig. 8C). Frequency of interictal spikes over 30 days post-seizure were significantly decreased in CD + shGAP43 rats at day 2 (p = 0.037) and day 30 (p = 0.0001) as compared to CD rats treated with scrambled shRNA after PTZ induced seizures (Fig. 8D). These results indicated that PTZ-induced epileptogenicity was inhibited in CD rats through GAP-43 knockdown. In contrast, frequency of interictal spikes in non-CD rats was very low and was not affected by GAP-43 knockdown (Fig. 8D).

## Discussion

We recently demonstrated that GAP-43 proteins are higher in the dysplastic and epileptic cortex compared to normal appearing cortex in human patients with medically intractable epilepsy who underwent surgical resections^14^. Using a rodent model of cortical dysplasia, we were able to further investigate this protein and its role in epileptogenesis. Our results show that a “second hit” (an induced seizure) in rats with CD leads to long-lasting (up to 120 days) upregulation of new synapse protein formation (GAP-43) in predominantly excitatory synapses. Persistent GAP-43 upregulation is correlated with the early development of epileptogenicity (interictal spikes) and ictogenesis (spontaneous epileptic seizures). Conversely, the knockdown of GAP-43 in CD brains decreased interictal spike frequency, further suggesting a direct role of GAP-43 in cortical dysplasia and its upregulation in epileptogenesis.

GAP-43 is an intracellular protein located at branching points, growth cones, and axon terminals involved in axonal elongation, neurotransmitter release and synaptic vesicle recycling^16,30^. It is activated through phosphorylation at ser41 via protein kinase C (PKC) leading to actin polymerization^31^. GAP-43 expression is crucial in neurodevelopment, yet it is significantly reduced in multiple brain areas (including sensorimotor cortex) after brain maturation. It remains abundant in plastic areas of the adult brain such as the limbic system, the hippocampus and associative areas of the brain, but is low in primary sensory and motor areas^15,16,32,33^. As GAP-43 is elevated during normal neurodevelopment, it is possible that the “first hit” (e.g. *in utero* irradiation model), causes GAP-43 expression to remain high in dysplastic cortex^14,16^. This could allow for the development of a highly plastic brain prone to synaptic outgrowth, potentially creating an imbalance between excitatory and inhibitory connections. GAP-43 protein expression is higher in the sensorimotor area of CD rats (as compared to control brains) with sustained upregulation up to older ages (~165 days postnatal). Animals with CD express high baseline GAP-43 levels and show a lower threshold for the development of longer and more severe PTZ-induced seizure than control rats with lower baseline GAP-43 proteins. Moreover, the abundant localization of GAP-43 proteins in excitatory synapses may suggest that baseline axon outgrowth is involved in decreased seizure threshold, as observed in CD rats.

Our findings demonstrate a relationship between epileptogenesis and upregulation of GAP-43 following a second hit in a model of cortical dysplasia. Acute seizures lead to an early and short-term upregulation of GAP-43 in both control and dysplastic brains. These results are consistent with previous studies showing the implications of GAP-43 protein regulation following stroke and traumatic brain injury, as well as neurodevelopment, learning and memory; thus, suggesting a role of GAP-43 in plastic events, even in adulthood^16–19,34–36^. GAP-43 proteins were significantly higher in CD rats in the acute period following the “second hit” and the upregulation only persisted beyond the acute period in CD rats (as compared to control rats). We have also previously observed an increased expression of GAP-43 in other seizure models, including focal intracortical penicillin injection, kainic acid, and traumatic brain injury^37^. Acute, non-sustained GAP-43 upregulation may be a non-specific response to an insult to the normal brain. However, persistent GAP-43 up-regulation may trigger abnormal synaptogenesis in a dysplastic brain, and lead to the development and progression of chronic epilepsy.

The localization of GAP-43 protein in excitatory synapses, in addition to heightened epileptic activity with increased GAP-43 expression suggest that axon outgrowth plays a role in the epileptogenesis in CD following a “second hit”. The sustained increase in GAP-43 aligns at least in part with results of surgical outcome studies^5,12,38–40^. The results of our confocal imaging show that GAP-43 positive synapses are primarily glutamatergic. These results could support the notion that there is a change in the balance of excitation and inhibition throughout epileptogenesis, and further suggests that GAP-43 upregulation is involved in this process. This data is consistent with research by Console-Bram *et al*. showing GAP-43 upregulation following exposure to glutamate, but a decrease with exposure to GABA^41^. With an increase in excitatory neurotransmission through glutamate receptors, GAP-43 may be further upregulated, creating more excitatory synapses and a potential positive feedback loop in epilepsy-prone brains. This pathway may begin with an excitatory stimulus, or heightened expression of an excitatory receptor such as NMDA, leading to an increase in GAP-43 with new excitatory synapses and a pro-epileptic network. Previous studies showed that NMDA receptors play a role in the regulation of GAP-43, and may serve as a potential up-stream target^42,43^. Additionally, upregulation of the NMDA receptor complex in human dysplastic neurons, contributes to the formation of local epileptic networks^44–48^. We have observed an increase in NMDA receptor 2 A/B subunits in CD brains as compared to non-CD, with GAP-43 positive axons surrounding the NR2A/B positive areas on the neuronal cell bodies^37^. Previous reports also showed that blocking NMDA receptors prevents the increased expression of GAP-43; making NMDA receptors an attractive upstream candidate for GAP-43 regulation^41,49^.

The potential role of GAP-43 in epileptogenesis after a second hit is further highlighted in CD animals following GAP-43 knockdown. The knockdown of GAP-43 reduced the peak seizure severity after PTZ induced acute seizures. Moreover, the reduction in interictal spike frequency following the acute seizure, or “second hit”, provides further evidence for a role of GAP-43 in epileptogenesis. Excitatory post-synaptic protein PSD-95 was significantly reduced with GAP-43 knockdown. This may contribute to the observed inhibition of chronic epileptogenesis. Further research is warranted to investigate the downstream targets that may play a role in modulation of a pro-epileptic neuronal network. The mechanisms involved in the increased expression of GAP-43 protein are likely multifactorial. For example, an intrinsic program-driven transcription of GAP-43 expression, supported by previously published results showing increased GAP-43 mRNAs in dysmorphic neurons^50,51^.

Levels of GAP-43 in the serum were elevated after spontaneous seizures, but not prior to their onset. The abrupt increase in GAP-43 levels in the serum is observed at day 120, but not day 100. Similarly, while interictal spikes continuously increase up to 120 days, we did not observe spontaneous seizures until 120 days after PTZ. There was also a correlation between serum GAP-43 levels and interictal spikes at days 100 and 120. This phenomenon must be further explored, and future studies should focus on protein changes at day 120 and later. We also observed (using immuno-EM) aggregated GAP-43 protein in the capillary lumen of CD + SS rats, but did not observe this phenomenon in other groups. These observations suggest that the post-ictal GAP-43 serum level increase could be due to leakage secondary to a seizure-induced breakdown of the blood brain barrier^26,27^.

In summary, our results indicate that the sustained increase of GAP-43 in dysplastic, but not in control brains, is involved in epileptogenesis and epileptogenicity. This rather specific expression of GAP-43 may point to a fundamental pathophysiological mechanism for expression of epileptogenicity and disease progression in epilepsy due to CD. We postulate that during the latent period, molecular/cellular changes, such as aberrant axonal sprouting, occur in the CD brain resulting in the development and growth of local, highly excitable and electrophysiologically synchronized epileptic network(s).

## Methods

### Animals

A total of twenty timed-pregnant Sprague-Dawley rats and their 113 male pups were used in this study (Charles River Laboratories, Wilmington, MA). The care and use of the animals were approved by the Animal Research Committee of Cleveland Clinic Foundation. All methods were performed in accordance with the approved guidelines by the Institutional Animal Care and Use Committee and the Institutional Biosafety Committee of Cleveland Clinic. All rats included in the study were housed in individual cages, under 12-hour dark-light cycles, and had free access to food and water. Animals were housed in the Cleveland Clinic Biological Resources Unit accredited by the Association for Assessment and Accreditation of Laboratory Animal Care International.

### *In utero* irradiation for a model of cortical dysplasia

Ten timed-pregnant rats were irradiated with 145 cGy of cesium-137 on E17 for a model of multifocal cortical dysplasia as previously described^20–22^. An additional ten timed-pregnant rats served as age-matched control animals. All pups were grown to adulthood of post-natal day 55 (PND55) prior to experimental procedures, which is when non-CD and CD animals weigh roughly the same weight, therefore reducing possible differences in drug metabolism.

### Characterizing GAP-43 expression after acutely generalized seizure

In order to account for potential molecular changes caused by surgical implantation of EEG electrodes, parallel groups of rats were studied with and without EEG. In the first set of experiments, 50 rats (25 non-CD, 25 CD) without surgical manipulations were injected with pentylenetetrazole (PTZ) at a sub-convulsive dose (40 mg/kg, i.p.). This subconvulsive dose only induces seizures in a small portion of normal/non-irradiated rats, but it leads to *status epilepticus* in the vast majority of CD rats^23^. Fifteen (9 non-CD, 6 CD) animals were injected with saline and used for normal and CD controls. All animals were monitored visually by video and given a behavioral score for seizure severity^25^. Rats were then sacrificed at post-PTZ induced seizure day 2, day 15, day 30, or day 120 and their brains were harvested for western blot and ELISA or fixed in paraformaldehyde solution for immunohistochemistry.

### Long-term EEG and video monitoring after acute generalized seizure

In the second set of experiments, 28 rats (PND55; 14 non-irradiated, 14 CD) were surgically implanted with EEG electrodes for monitoring of epileptic activity over time. These animals were also evaluated for GAP-43 expression, but since GAP-43 may increase due to the acute damage involved in surgery, the initial experiments were also necessary to confirm that the surgical procedures were not the cause of the changes in expression. Under isoflurane anesthesia, rats were placed in a stereotactic frame and implanted with six epidural EEG electrodes as described in Nemes *et al*., 2016. Electrodes were implanted bilaterally over the frontal (LF: left frontal cortex, RF: right frontal cortex, A: +1.0 mm, L: ± 2.5 mm from bregma) and parietal cortices (LP: left parietal cortex, RP: right parietal cortex, A: −3.6 mm, L: ± 2.5 mm from bregma). Additionally, two reference electrodes were placed REF1: above the frontal sinus +3.0 mm anterior and 1.5 mm lateral to bregma and REF2: above the cerebellum −2.0 mm posterior and 2.5 mm lateral to lambda for a back−up reference electrode. After 72 hours of recovery, rats were placed in EEG recording chambers. They were injected with either PTZ or saline, in the same manner as described above. Rats were monitored in 12-hours per day, three to four days per week, for up to 120 days (mean total hours per rat = 623.14 ± 144.9; 43.57 ± 2 hours per animal each week). Four rats were monitored simultaneously during each session at an acquisition sampling rate set at 200 Hz, a low-frequency filter setting of 7 Hz and high-frequency filter of 70 Hz. Animals had free access to food and water during the recording sessions.

### Investigating the effects of GAP-43 knockdown on epileptogenesis

In a third experiment, 20 rats (PND55; 10 non-irradiated, 10 CD) received an intracortical injection of a lentiviral vector (LTV) containing shRNA against GAP-43 (shGAP43, 7 non-CD, 7 CD), or a control vector containing a scrambled sequence of the GAP-43 shRNA (shScramble, 3 non-CD, 3 CD). Lentiviral vectors^52^ were purchased from Sigma-Aldrich, Inc. (St Louis, MO) at a titer of 10^9^ TU/ml. Lentivirus injection was conducted in a biosafety level 2 laboratory within a biosafety cabinet. Rats were injected during surgical implantation of EEG electrodes under isoflurane anesthesia. Using stereotactic technique as described above, a burr hole was created at A/P: −2.0 mm L: +3.0 mm D: 1.0 mm. A Hamilton syringe (Hamilton Company, Franklin, Massachusetts) was loaded with 2 µl of lentiviral suspension and placed into the cortex. After 2 minutes, infusion of the lentivirus began, with a rate of 0.1 µl per minute. After 2 µl of the lentivirus was injected, the needle remained in place for 5 minutes to ensure proper infusion. Afterwards, it was slowly removed and the burr hole was closed with dental acrylic.

Six days after LTV injection, rats began EEG monitoring with a baseline recording of 24 hours. At day 7 post-LTV, rats were given 10 mg/kg, i.p., of pentylenetetrazole (PTZ) every 10 minutes until an EEG seizure was observed to determine their seizure threshold. Seizure severity was graded based on duration and clinical semiology using Racine’s behavioral scale^25^.

### EEG and Video Analyses

Digitized EEG was reviewed using NeuroWorkbench software (Nihon Kohden America Inc., Irvine, CA, USA). EEG signals were reformatted in referential and bipolar montages. All EEG and video was visually inspected for the presence of seizures, and 25% of EEG was sampled for manual quantification of interictal spikes (3 hours per 12 hour recording). Analyses were performed by two blinded investigators (AN, ZY). The EEG seizures were defined as repetitive and evolving spikes with interruption of the background activity for 10 seconds or longer. As previously reported, epileptiform/interictal spikes were defined as paroxysmal electrical sharp activities lasting 20–150 ms, with an amplitude that was at least five times the background EEG activity^21,22^. Seizure and spike data were analyzed based on latency from PTZ injection, frequency, and severity.

### Histology

Once animals completed their monitoring periods, they were deeply anesthetized with ketamine (100 mg/kg, i.p.) and xylazine (10 mg/kg, i.p.) and perfused transcardially with either phosphate-buffered saline (PBS) or 4% paraformaldehyde (PFA). Their brains were then harvested and either flash-frozen in liquid nitrogen, or stored in 4% PFA for 24 hours and then transferred into 30% sucrose for at least 48 hours before processing. The brains were serially sectioned on the coronal plane in 30 µm slices using a cryostat. The slices were stained with Cresyl Violet (CV) for histological examination. Slides were also used to confirm the CD as previously described^23^.

Immunohistochemistry (IHC) and immunofluorescence (IF) staining was done on free-floating sections^53^. Slices were incubated overnight at 4 °C in the following primary antibodies: anti-GAP43 (1:1000, rabbit polyclonal; Sigma-Aldrich, Inc., St Louis, MO) or anti-GAP43 (1:200, mouse monoclonal; Invitrogen, Thermo Fisher Scientific, Waltham, MA) combined with anti-Vesicular Glutamate Transporter 1 (VGLUT1, 1:50, mouse monoclonal; Synaptic Systems, Goettingen, Germany) or anti-Vesicular GABA Transporter (VGAT, 1:50, mouse monoclonal; Synaptic Systems, Goettingen, Germany). IHC staining was developed using VECTASTAIN Elite ABC Kit (Rabbit IgG) (Vector Laboratories, Burlingame, CA) according to manufacturer’s instructions. IF staining was developed using Alexa Fluor^®^ 488 goat anti-mouse and 568 goat anti-rabbit secondary antibodies according to manufacturer’s instructions (1:15,000, goat polyclonal; Invitrogen, Thermo Fisher Scientific, Waltham, MA). As a negative control used to confirm the absence of non-specific staining, the method was also completed without primary antibodies. In addition, antibody specificity was confirmed using pure GAP-43 protein with western blotting and ELISA. Positive signals were observed with purified protein, and western blotting bands were located only at 43 kDa.

### Microscopy

Slides were imaged using a confocal laser scanning microscope (TSC SP5; Leica Microsystems, Wetzlar, Germany) at 1024 × 1024 with a 40x lens. Z stacks were gathered from each sample with 1 µm step sizes throughout a total thickness of 100 µm. Two slides per animal were analyzed with one image taken from each hemisphere of the somatosensory cortex (4 images per rat total). Once the settings for laser intensity, signal offset and amplifier gain were calibrated, they were left the same for all slides. Background was subtracted and monochromatic images were analyzed for co-localization using the Intensity Correlation Analysis plugin (ImageJ v1.37a) and positive product of the differences from the mean (PDM) as described^54,55^. A Pearson’s correlation was used to compare the relationships of each antibody pair. Image stacks were also analyzed for pixel intensity by each color and data was analyzed using Students t-tests. For immuno-electron microscopy (EM), brains were processed as described by Fujioka *et al*., 2011 for pre-embedding immuno-EM. Thin sections were blocked using bovine serum albumin and incubated in anti-GAP43 (1:1000, rabbit polyclonal; Sigma-Aldrich, Inc., St Louis, MO) for 12 hours at 4 °C. Sections were washed and incubated for 1 hour in 10-nm gold conjugated goat anti-rabbit IgG (Ted Pella, Inc., Redding, CA) and fixed to stabilize gold particles for imaging with electron microscopes (Zeiss CEM902, Oberkochen, Germany; JEOL 1200EX, Tokyo, Japan).

### Molecular Biology

Brains were homogenized and prepared for RNA and protein quantification as described^56^. Total RNA was isolated from LTV brain samples using TriZol reagent. Reverse transcription was done with the High-Capacity cDNA Reverse Transcription Kit, and quantitative real-time PCR was completed with a 7500 Fast Real-Time PCR System instrument using Taqman Fast Universal PCR Master Mix (2X), No AmpEraseUNG (all from Applied Biosystems, Foster City, CA). Probes and primers were from Taqman gene expression assay reagents: GAP-43 (Hs00967138_m1). Data were normalized to GAPDH (Hs02758991_g1) RNA amplification and calculated relative to the expression of the target gene in the brains of LTV rats.

For increased sensitivity and quantification of protein expression, NIR western blotting technique was used. Protein concentrations adjusted to exactly 0.5 μg were electrophoresed and transblotted onto Millipore Immobilon^®^-FL polyvinylidene fluoride (PVDF) membrane (LI-COR Biotechnology, Lincoln, NE, USA). Membranes were blocked with Odyssey Blocking Buffer (TBS) and incubated with anti-GAP43 (1:2000, rabbit polyclonal, Sigma-Aldrich, Inc., St Louis, MO, USA), anti-GAPDH (1:1000, mouse monoclonal, Thermo Fisher Scientific, Waltham, MA, USA), PSD-95 (1:500, mouse monoclonal, EMD Millipore, Billerica, MA, USA) or Gephyrin (1:200, rabbit polyclonal, Santa Cruz Biotechnology, Dallas, TX, USA) overnight at 4 °C. Membranes were washed thoroughly in TBS-t and incubated in secondary antibodies for 1 hour (1:15000, IRDye 800 CW Goat anti-Rabbit IgG; 1:15000 IRDye 680RD Goat anti-Mouse IgG, LI-COR Biotechnology). After drying overnight, membranes were scanned on the Odyssey^®^ Classic Imager (84 µm resolution, medium dynamic range, 0.0 nm height, intensity 4, channels 700 & 800). Signals were quantified and normalized using methods to eliminate background as well as determine relative signal over total protein, and then expressed as fold changes over the signal of the control group^57,58^.

A sandwich ELISA method was used as previously described to measure the concentration of GAP43 in either the brain homogenates or serum^59^. ELISA plates were coated with monoclonal anti-GAP-43 antibody (Invitrogen, Thermo Fisher Scientific, Waltham, MA) at the concentration of 1 µg/ml in ELISA coating buffer (Bicarbonate/carbonate buffer) overnight. Plates were then blocked with 3% BSA for two hours at room temperature and washed with PBS +0.05% tween. Diluted brain homogenates (100 ng/mL), serum (1:100 dilution) or purified GAP 43 (Invitrogen, Thermo Fisher Scientific, Waltham, MA) at varying concentrations (1–1000 ng/ml) as standards were added overnight at 4 °C. Following washes, biotin conjugated polyclonal anti GAP43 antibody (Thermo Scientific, Waltham, MA) was added (1 µg/ml) and plates were incubated for one hour at room temperature. Plates were then washed and incubated with streptavidin HRP for one hour at room temperature, and developed using ABTS peroxidase substrate system. Plates were read on an ELISA reader at 410 nm. Using standards, known concentrations, their optical density (OD), and the optical density of samples, linear regression curves were constructed and the concentration of GAP43 was calculated in the samples using the equations from the linear regression line.

### Statistical Analyses

Statistical analyses were performed using IBM SPSS Statistics for Windows, Version 22.0 (IBM Corp., Armonk, NY, U.S.A.) and R Statistical Software (version 2.14.0; R Foundation for Statistical Computing, Vienna, Austria). Comparisons were made between groups based on seizure prevalence, latency and duration. Seizure severity was graded behaviorally using a standard scale^25^ and compared with a mixed models regression analyses with Tukey-Kramer adjustments. Severity was also evaluated by percentage of time spent in each stage over the total seizure duration. GAP-43 protein expression from IHC was graded as previously described^14^. Mixed models regression analyses were run on all numeric data comparing CD and Non-CD across groups of control, day 2, day 15, day 30 and day 120 time points. Students t-tests were used to evaluate independent groups when appropriate. Results were considered statistically significant with p-values < 0.05.

## Electronic supplementary material

Supplementary Raw Image for Figure 8

Supplementary Raw Image for Figure 3
