# Supplementary figures and images for: Growth Associated Protein 43 (GAP-43) as a Novel Target for the Diagnosis, Treatment and Prevention of Epileptogenesis
Source: Sci Rep. 2017 Dec 18;7:17702. doi: 10.1038/s41598-017-17377-z (PMC5735087; doi:10.1038/s41598-017-17377-z)

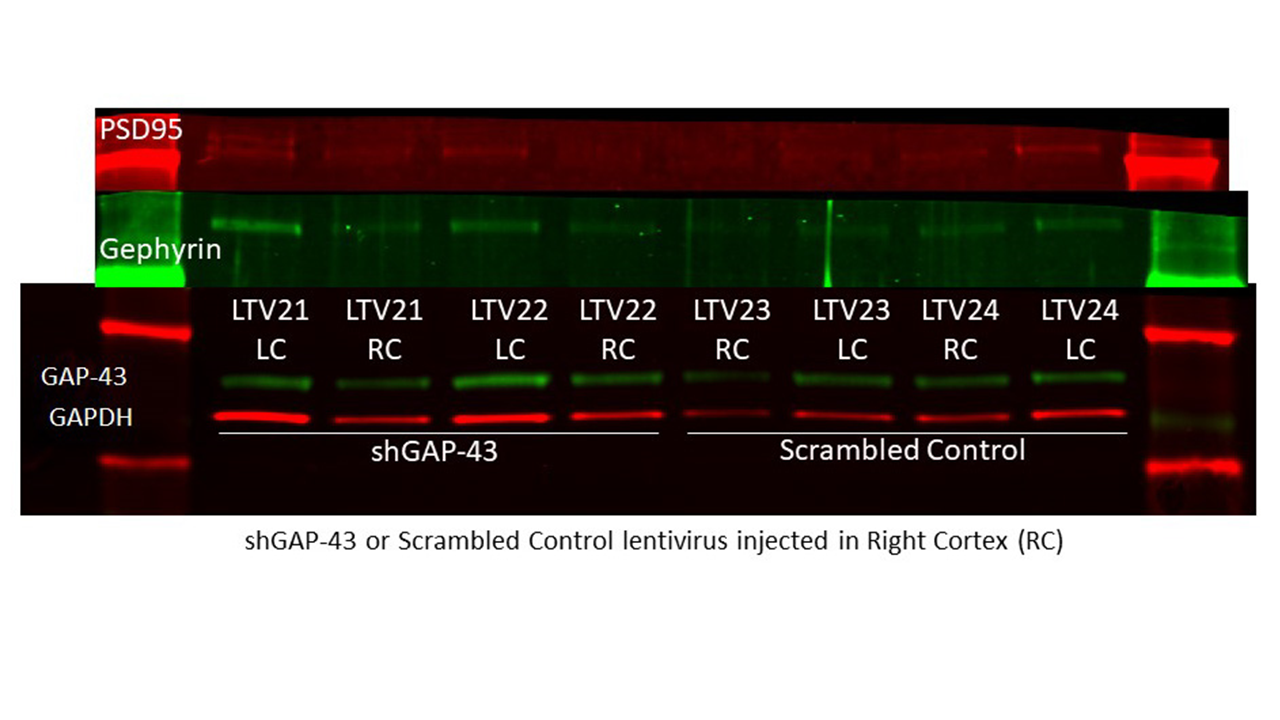

Supplement: Supplementary file 1 — Supplementary Raw Image for Figure 8 [file 41598_2017_17377_MOESM1_ESM.tif]

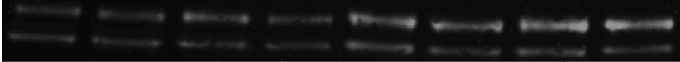

Supplement: Supplementary file 2 — Supplementary Raw Image for Figure 3 [file 41598_2017_17377_MOESM2_ESM.tif]
